# Whey protein isolate-gelatin nanoparticles enable the water-dispersibility and potentialize the antioxidant activity of quinoa oil (*Chenopodium quinoa*)

**DOI:** 10.1371/journal.pone.0240889

**Published:** 2020-10-30

**Authors:** Keith Hellen Dias da Silva Lira, Thaís Souza Passos, Heryka Myrna Maia Ramalho, Karla Danielly da Silva Ribeiro Rodrigues, Érica de Andrade Vieira, Angela Maria Tribuzy de Magalhães Cordeiro, Bruna Leal Lima Maciel, Karla Suzanne Florentino da Silva Chaves Damasceno, Francisco Canindé de Sousa Júnior, Cristiane Fernandes de Assis

**Affiliations:** 1 Nutrition Postgraduate Program, Center for Health Sciences, Federal University of Rio Grande do Norte, Natal, RN, Brazil; 2 Department of Nutrition, Center for Health Sciences, Federal University of Rio Grande do Norte, Natal, RN, Brazil; 3 Biotechnology Postgraduate Program, Health Science, Potiguar University, Natal, RN, Brazil; 4 Food Science and Technology Postgraduate Program, Technology Center, Federal University of Paraiba, João Pessoa, PB, Brazil; 5 Department of Pharmacy, Center for Health Sciences, Federal University of Rio Grande do Norte, Natal, RN, Brazil; VIT University, INDIA

## Abstract

The quinoa oil presents benefits to health, but its low water dispersibility in the aqueous matrix and instability of bioactive compounds is challenging for food application. This study performed the physicochemical and chemical characterization of quinoa oil and evaluated its water dispersibility and 2,2’-azinobis-(3-ethylbenzothiazoline-6-sulfonic acid) radical scavenging activity after nanoencapsulation in porcine gelatin and combination with whey protein isolate by emulsification O/W technique. Thus, three formulations were obtained: 1) OG–containing quinoa oil and porcine gelatin in aqueous phase 2; 2) OWG1—containing quinoa oil, whey protein isolate, and porcine gelatin in aqueous phase 2; and 3) OWG2—containing quinoa oil and whey protein isolate in aqueous phase 1, and porcine gelatin in aqueous phase 2. The oil characterization showed that quinoa oil presented the predominance of linoleic acid (53.4%), and concentration of alpha and gamma-tocopherol, respectively, of 8.56 and 6.28 mg.100g^-1^. All formulations presented a smooth surface without depression or cracking, an average diameter between 165.77 and 529.70 nm. Fourier transform infrared spectroscopy indicated chemical interaction between the encapsulating agents and the oil in all formulations, being more intensified in OWG1 and OWG2. Based on this, these formulations showed higher dispersibility in aqueous solution [68% (3.48) and 71% (2.97)]. This resulted in higher antioxidant activity for OWG1 and OWG2, showing the amounts that reduces antioxidant activity by 50% equal to 5.30 (0.19) mg/mL and 5.54 (0.27) mg/mL, respectively, compared to quinoa oil [13.36 (0.28) mg/mL] (p < 0.05). Thus, quinoa oil nanoencapsulation proved to be an efficient alternative to enable water-dispersibility and enhance antioxidant activity, increasing its potential for application in the food industry.

## Introduction

Quinoa (*Chenopodium quinoa* Willd.) is a plant native from the Andean region that grows at many different altitudes and under various climatic conditions, being an excellent pseudocereal due to its nutritional characteristics [1]. Therefore, it has been outstanding due to excellent nutritional, characterized by the high biological value proteins, bioavailable essential amino acids, unsaturated lipids, dietary fibers and other bioactive compounds (ex. phenolic compounds) [2].

It is considered an alternative oilseed, due to the quality and quantity of the lipid fraction present in its constitution. Quinoa oil has a high degree of unsaturation, being rich in essential fatty acids, such as linoleic and α-linolenic [3]. It also stands out for having a significant concentration of natural antioxidants such as gamma and alpha-tocopherol [4].

The growing interest of consumers in quinoa is related to issues involving nutritional benefits, which leads the food industry to increase interest in developing products with a high nutritional value capable of providing benefits to human health [5]. Thus, the nutrient composition of quinoa oil favors the study for an application in an aqueous food matrix. However, due to its lipophilic nature, and bioactive compounds instability to the exposure of heat, light, and oxygen, represent technological challenges hinder its use by the food industry [6].

In this context, the nanoencapsulation techniques offer solutions to improve the oil dispersibility in an aqueous food matrix and bioavailability of bioactive compounds [7]. Jungret et al., [8] reinforce that it is an interesting and efficient strategy to improve the physical stability of bioactive compounds, aiming to protect them from possible interactions with the other ingredients present in food matrices. Besides, the subcellular size can promote an increase in the bioactivity of the bioactive. As far as we could determine, there are no studies involving the nanoencapsulation of quinoa oil, and this was the goal of this study.

Emulsification is one of the most widely used encapsulation techniques to promote the encapsulation of vegetable oils, which involves obtaining oil-in-water (O/W) emulsions, for example, when small drops of oil are dispersed in aqueous solution. This allows the protection of the bioactive and promotes controlled release at the desired time and place, in addition to masking undesirable flavors [9]. In this context, Zhang et al. [10] pointed out in a review study that one of the advantages of this technique is to use the emulsions obtained in fluid or solid form. With this, the powder forms can be obtained by drying the fluid emulsion by lyophilization or atomization. Thus, the powders obtained have numerous applications in the areas of food and pharmaceuticals.

Numerous encapsulating agents can be used to promote the encapsulation of vegetable oils. Ghadermazi et al. [11] highlight that the whey proteins have various critical functional properties for the food industry. Emulsification, gelling, and stabilization are interesting to promote the obtaining of formulations containing encapsulated bioactive compounds. Whey protein components, such as beta-lactoglobulin (β-LG, ∼50%) and alfa-lactalbumin (α-LA, 58 ∼20%), exhibit an affinity to a wide of bioactive components [12].

According to the review study by Kharim and Bath [13], gelatin is another macromolecule that combines interesting functional properties to be used as an active surface agent and wall material. It is a low-cost material obtained from collagen hydrolysis, mainly from porcine (Type A) and bovine (Type B), that presents several applications in the food, biomedical, and pharmaceutical industries. Gelatin can be classified as Type A (isoelectric point—pH 8 and 9) or B (isoelectric point—pH 4.5 and 5.6), depending on the type of treatment used to obtain it.

Based on this, the present study aimed to perform the physicochemical and chemical characterization of quinoa oil (*Chenopodium quinoa* Willd) as well as the production and characterization of quinoa oil encapsulated in porcine gelatin and combination with whey protein isolate, using oil/water and multilayered emulsification as encapsulation techniques to increase the quinoa oil solubility in water.

## Materials and methods

### Materials

The quinoa oil of the species *Chenopodium quinoa* Willd was donated by the company Plantus® S.A. RN/Brazil. Alpha-tocopherol, gamma-tocopherol, porcine gelatin (Type A) and Tween 20 were obtained from Sigma Aldrich®, while the whey protein isolate was obtained from Alibra®. As measured by the Biuret method, the protein content of porcine gelatin was 80% (w/w). And for whey protein isolate was 88% (w/w) according to the manufacturer.

### Determination of fatty acids profile of quinoa oil

Esterification to obtain fatty acid methyl esters (FAMEs) was performed, following the methodology described by Hartman and Lago [14]. The quantification was performed by normalization of the peak areas and the identification by the mass Spectra Database Library (NIST), using a GCMS-QP2010 (Shimadzu, Kyoto, Japan) equipped with a Durabound DB-23 column (30 mm x 0.25 mm x 0.25 μm). The injection port and detector temperature were fixed at 230°C while the column temperature was set at 90°C. The elution gradient in the column was 90 to 150°C (10°C/min), 150 to 200°C (2°C/min), 200 to 230°C (10°C/min) in a total run of 39 minutes with a split of 100. The carrier gas was He [15].

### Physicochemical characterization of quinoa oil

Physicochemical characterization of quinoa oil was established by the following parameters: acidity index (AI), peroxide index (PI), saponification index (SI), iodine index (II), refraction index (RI) and relative density, performed according to the [16].

### Quantification of alpha and gamma-tocopherol in quinoa oil using High-Performance Liquid Chromatography (HPLC)

Alpha and gamma-tocopherol identification and quantification were performed according to Grilo et al. [17] with modifications. 200 μL of quinoa oil was diluted in 800 μL of dichloromethane and applied through direct injection, according to Lima and Gonçalves, (1997). Sample preparation was done in temperature around 25°C, using tubes protected from light (covered with aluminum foil).

50 μL of each sample was injected into a chromatograph (Shimadzu), with LC-10 AD (Shimadzu) pump coupled to an SPD-10A (Shimadzu) UV-VIS Detector and Chromatopac C-R6A (Shimadzu) integrator with an Ascentis® C18 (Sigma Aldrich®) 25 cm x 4.6 mm column. Methanol (50%) and acetonitrile (50%) were the mobile phase, in an isocratic system, with a flow rate of 1.0 mL.min^-1^. The identification and quantification of alpha and gamma-tocopherol in the samples were established by comparison with the retention time and the area of the respective standards (alpha-tocopherol and gamma-tocopherol). The specific extinction coefficient confirmed the standard concentrations (ε 1%, 1 cm = 75.8 with a wavelength of 292 nm for alpha-tocopherol and ε 1%, 1 cm = 91.5 with a wavelength of 298 nm for gamma-tocopherol), both diluted in absolute ethanol [18].

### Quinoa oil encapsulation

#### Nanoparticles loaded with quinoa oil

The particles were formulated using porcine gelatin and a combination of porcine gelatin and whey protein isolate (WPI) as encapsulating agents, Tween 20 as a surfactant, and quinoa oil.

Based on bench standards were obtained formulations by oil/water (O/W) emulsification technique through the homogenization of the oily phase with the aqueous phase 1 (90 mL), followed by the homogeneization of the aqueous phase 2 (100 mL) at 17,000 rpm/10 min, according to Medeiros et al. [19] with modifications. Thus, three groups were obtained: 1) Quinoa oil and porcine gelatin in aqueous phase 2 (OG); 2) Quinoa oil, whey protein isolate and porcine gelatin in aqueous phase 2 (OWG1); 3) Quinoa oil, whey protein isolate in aqueous phase 1, and porcine gelatin in aqueous phase 2 (OWG2) (Table 1).

**Table 1:** Formulations produced to obtain quinoa oil loaded particles.

Formulations	Oil phase (quinoa oil) (g)	Aqueous phase 1 (90 mL)		Aqueous phase 2 (100 mL)		
		Tween 20 (% w/v)	Whey protein isolate (% w/v)	Tween 20 (% w/v)	Porcine gelatin (% w/v)	Whey protein isolate (% w/v)
**OG**	4	1.5	-	1.5	4.0	-
**OWG1**	4	1.5	-	1.5	3.0	1.0
**OWG2**	4	1.5	1.0	1.5	3.0	-

OG: Quinoa oil and porcine gelatin in aqueous phase 2; OWG1: Quinoa oil, whey protein isolate and porcine gelatin in aqueous phase 2; OWG2: Quinoa oil, whey protein isolate in aqueous phase 1, and porcine gelatin in aqueous phase 2.

For the preparation of aqueous phase 1 of all formulations, magnetic agitation was used for 30 minutes at room temperature to solubilize the materials. For the OWG2 formulation, after the agitation period, the pH of the solution was adjusted to 5.5 using HCl.

The aqueous phase 2 was prepared in two steps. The first step for all tested formulations consisted of solubilizing the porcine gelatin in distilled water for 1 hour at 40°C, under magnetic agitation. For the second step, Tween 20 (OG, OWG2) or WPI and Tween 20 (OWG1) were solubilized under magnetic agitation during 40 minutes at room temperature. Subsequently, the solutions produced were homogenized, and the pH was adjusted to 5.5 with HCl PA. Then, homogenized again under magnetic stirred at room temperature for 40 minutes.

The oily phase, composed of quinoa oil, was homogenized to the aqueous phase 1 using ultradisperser (Ultra-Turrax, IKA® T18 Basic) at 17.000 rpm/10 minutes. Then, the aqueous phase 2 was homogenized to the first emulsion using the same conditions.

The production was performed in triplicate, and the obtained emulsions were dried by freeze-drying (LioTop L101) at -57°C and pressure of 43 μmHg. The powder encapsulates were triturated in a blender and stored under freezing (-18°C) for further analyses.

### Particle characterization

#### Scanning Electron Microscope (SEM)

SEM was performed to determine the different formulations morphology and physical size. The particles were suspended in acetone and then dripped into silicon plates fixed in stubs. The analysis used a MEV-FEG ZEISS microscope (AURIGA), in different magnifications, with a high vacuum, 2–3 kV voltage, and no metallization.

#### Laser diffraction

5.5 mg of the OG and 10 mg of the other lyophilized formulations were dispersed in acetone under magnetic agitation at room temperature for 2 minutes. Subsequently, based on Medeiros et al. [19] with modifications, formaldehyde PA was added to the dispersions (2 mL for formulations OG and OWG1, and 3 mL for OWG2), to deagglomeration and facilitate particle size analysis. The dispersions were stirred at different times: 10 minutes for OG and OWG1, and 30 minutes for OWG2.

Then, the dispersions were filtered, and the particle mass retained in the qualitative filter paper was collected for laser diffraction. The materials were re-dispersed in 4 mL of acetone. Tests in the laboratory standardized all the variables of this process.

The dispersions were analyzed at 5 runs/1 min in the NanoBrook ZetaPlus Zeta potential Analyzer, Brookhaven Instruments Software–ZetaPALS Particle Sizing Software, for measuring the average diameter and the polydispersity index. The experiments and measurements were performed in triplicate.

#### Zeta potential

For the analysis of the Zeta Potential, 10 mg of each encapsulated were dispersed in 4 mL of distilled water, then disposed in acrylic cuvettes with lateral electrodes. A total of 10 runs, 1 minute each, were performed in the NanoBrook ZetaPlus potential Analyzer, coupled with the Software Brookhaven Instruments–PALS Zeta potential Analyzer. The experiment was performed in triplicate.

#### Fourier Transform Infrared Spectroscopy (FTIR)

All formulations, porcine gelatin, whey protein isolate, Tween 20, and quinoa oil were analyzed by FTIR. The materials were homogenized with potassium bromide (KBr), macerated, and pressed to obtain tablets. Subsequently, the samples were analyzed in transmittance and with a medium infrared region of 4000 to 400 cm^-1^. A Shimadzu spectrometer, model FTIR-8400S, IRAFFINITY-1 series, IRSOLUTION software, version 1.60, with a scan number of 32 and resolution of 4 cm^-1^ was used.

#### X-ray Diffraction (XRD)

All formulations and the encapsulating agents (PG and WPI) were analyzed in a high-resolution X-Ray diffractometer (SHIMADZU, XRD 7000 model) with Seifert generator ID3000. The materials were placed in cylindrical samples holder and analyzed at diffraction angle 2 ɵ between 0 and 50°.

#### Total quinoa oil determination

Total quinoa oil determination (dry basis) in each formulation was performed according to González et al. [20] with modifications. The quinoa oil (QO) was extracted using Soxhlet’s method. The result was expressed in percentage according to the following equation: QO (%) = quinoa oil present in powder formulation (g) / total quinoa oil used in the process (g) x 100. The analyses were performed in triplicate, and the values were expressed as mean and standard deviation.

#### Water dispersion assay

The water dispersion assay was performed according to Eastman & Moore [21] with modifications. A total of 100 mg of the encapsulated and 200 mg of crude quinoa oil were weighed and dispersed into 4 mL of distilled water in test tubes. The tubes were taken to orbital agitator (QUIMIB®—Q816M20) for 48 hours at 27°C (± 2°C) and 120 rpm. The analyses were performed in triplicate.

After 48 hours, the material was centrifuged (CENTRIBIO) at 3000 x g for 5 minutes. The lipid fraction was separated from the solubilized fraction and transferred into previously weighed porcelain capsules and was dried in an oven at 105°C for 5 h, then weighed to determine the amount of non-solubilized material. The total encapsulated material (dry basis) was subtracted from the non-solubilized content to determine the solubilized amount. The result was expressed in percentage.

#### Antioxidant activity

The antioxidant activity was evaluated by ABTS• radical scavenging activity. The radical ABTS was prepared, according to Rufino et al. [22]. Quinoa oil and formulations were prepared based on Rakmai et al. [23] with modifications in DMSO proportion. 60 μL of each group (QO, OG, OWG1, and OWG2) in the concentrations of 1 mg/mL—15 mg/mL with 240 μL of an ethanol solution of ABTS were used. The absorbances were measured at 734 nm in an Elisa microplate reader (Hexasystens, Power Wave).

The inhibition percentage of the ABTS• radical was determined as described in equation 1. The linear regression curve was determined to calculate the concentration of the evaluated groups necessary to provide 50% of radicals scavenging activity (IC50), according to Poojary et al. (2015).

$$ABTS•\mathrm{radical}\;\mathrm{Inhibition}\;(\%)=100\;x\frac{\mathrm{Abs}\;\mathrm{control}-\mathrm{Abs}\;\mathrm{sample}}{\mathrm{Abs}\;\mathrm{control}}$$

### Statistical analysis

The results were expressed as mean and standard deviation. ANOVA and Tukey’s post-hoc test were used to compare the total QO in particles, the dispersion percentages of QO and formulations in water, and the results of antioxidant activity for QO and formulations evaluated. A significance level of 5% (p < 0.05) was used to determine a significant difference between the evaluated formulations. Statistical analysis was performed using Graph Pad Prism software version 5.0.

## Results and discussion

### Fatty acids profile

A predominance of linoleic fatty acid (55.39%) was observed (Table 2) in quinoa oil. This amount was similar to that found by Pellegrini et al. (2018)-(48.76–53.94%) and Chen et al. (2019) (55.20–62.80%). The results demonstrate that regardless of the extraction method used, linoleic acid (Omega 6) is predominantly in quinoa oil.

**Table 2:** Fatty acids profile of quinoa oil (*Chenopodium quinoa Willd*.) determined by gas chromatography of fatty acid methyl esters.

Fatty acids	%
C10:0 (capric acid)	ND*
C12:0 (Lauric acid)	ND*
C14:0 (Myristic acid)	0.68
C16:0 (Palmitic acid)	24.20
C16:1 (Palmitoleic acid)	0.53
C17:0 (Heptadecanoic acid)	ND*
C18:0 (Stearic acid)	2.15
C18:1n-9c (Oleic acid)	15.52
C18:1n-9t (Elaidic acid)	0.81
C18:2n-6c (linoleic acid)	55.39
C18:2n-6t (linolelaidic acid)	0.72
C18:3n-3 (α-linolenic acid)	ND*
C20:0 (Arachidic acid)	ND*
C20:1n-9 (Gandoic acid)	ND*
C22:0 (Behenic acid)	ND*
Saturated FattyAcids	27.03
Monounsaturated Fatty Acids	16.86
Polyunsaturated Fatty Acids	56.11

*Not detected.

The presence of 56.11% of poly-unsaturated fatty acids (PUFAs) in quinoa oil demonstrates its potential benefit to human health, such as effects on cholesterol reduction, Diabetes Mellitus (DM) prevention, and reducing the risk of cardiovascular disease [24].

Oleic acid was 15.52% of fatty acids, similar to the values reported in different studies with quinoa oil, which showed values from 18.60 to 29.84% [25, 26].

The absence of linolenic acid (omega 3) was observed, which does not reflect the results found in the literature, in which omega-3 percentages were from 3.60 to 9.10% [25, 26]. This result may be related to the oxidation of omega 3 during the extraction process. In assessing the oxidative stability of vegetable oils with different fatty acid compositions, Li et al. (2013) observed that PUFAs were easily attacked by free radicals that react with their double bonds and promote the formation of products such as short-chain aldehyde. Thus, a decrease of them was observed, and omega 3 was the most affected fatty acid.

The percentage of saturated fatty acids found in quinoa oil was 27.03%, especially palmitic acid (24.20%). This fatty acid was also the primary saturated fatty acid found in other studies with quinoa oil, however, in lower percentages [25, 26]. Chen et al. [27] analyzing 28 different varieties of quinoa, found an inversely proportional relationship between the concentration of oleic acid and saturated fatty acids. This relationship was confirmed in the present study since the evaluated quinoa oil showed a lower level of oleic acid and a higher concentration of palmitic acid. The trans-elaidic and linolelaidic fatty acids were observed in quinoa oil, however, in insignificant amounts when compared to the Codex Alimentarius [28].

#### Physicochemical properties

The acidity index [0.90 (0.14) mg.KOH^-1^] and peroxide value [2.75 (0.39) meq.Kg^-1^)] were within the maximum limits established by the Codex Alimentarius Commission [28] of 4.0 mg KOH.g^-1^, and 15 meq.Kg^-1^, respectively, for cold-pressed and unrefined vegetable oils.

According to Pinho & Souza [29], acidity is directly related to the quality of the raw material, to the processing and, mainly, to the conservation conditions of vegetable oils. The results obtained for the acidity index indicate that the studied quinoa oil showed satisfactory quality from the raw material to the preservation of the final product.

Iodine was 102.87 (0.81) cg I_2_g^-1^, inferior to that reported by Repo-Carrasco et al. (2003)—(127.81 cgI_2._g^-1^). The iodine value is the unsaturation measurement of oil [30] and explains why each oil presents a unique range of this value. Considering that the higher the oil unsaturation, the higher its iodine value, this result is consistent, because a percentage of 72.92% for unsaturated fatty acids was observed. This result is in accordance with Repo-Carrasco et al. (2013), who found 82.70% of unsaturated fatty acids.

The refraction index increases along with the number of unsaturations in the carbonic chain, it is correlated with the iodine value, and therefore widely used to identify vegetable oils [31]. The refraction index obtained for quinoa oil was 1.47 (0.00), the same refraction index found by Repo-Carrasco et al. [32].

Vegetable oils density and fats are inversely proportional to the chain length and directly proportional to the unsaturation degree of the fatty acids that are part of its composition [33]. According to Abollé et al. [34] the density in oils and fats varies from 900 to 930 kgm^−3^. The value found in the studied quinoa oil was within this range [916 (0.00) kgm^−3^]. However, no density values for quinoa oil were found in the literature.

### Quantification of alpha and gamma-tocopherol

Higher concentrations of vitamin E were found in the present study, of 8.56 (0.18) and 6.28 (0.25) mg.100g^-1^ oil for α-tocopherol and γ-tocopherol, respectively. These results may be related to the analysis by direct injection, while the other studies accomplish a previous saponification step. Ryan et al. (2007) [35] analyzing quinoa seeds, found 2.10 mg.100g^-1^ of α-tocopherol and 3.10 mg.100g^-1^ of γ-tocopherol.

Tocopherols concentration in vegetable oils can be determined by two methods: dilution of the sample in appropriate solvent and its direct injection in a chromatograph; or saponification of the sample for posterior extraction of the unsaponifiable fraction containing vitamin E [36]. The dilution and direct injection of the sample is a faster and simpler method that reduces the possibility of sample degradation. Saponification is more time-consuming and increases vitamin degradation.

### Characterization of the encapsulates containing quinoa oil

**Fig 1:**
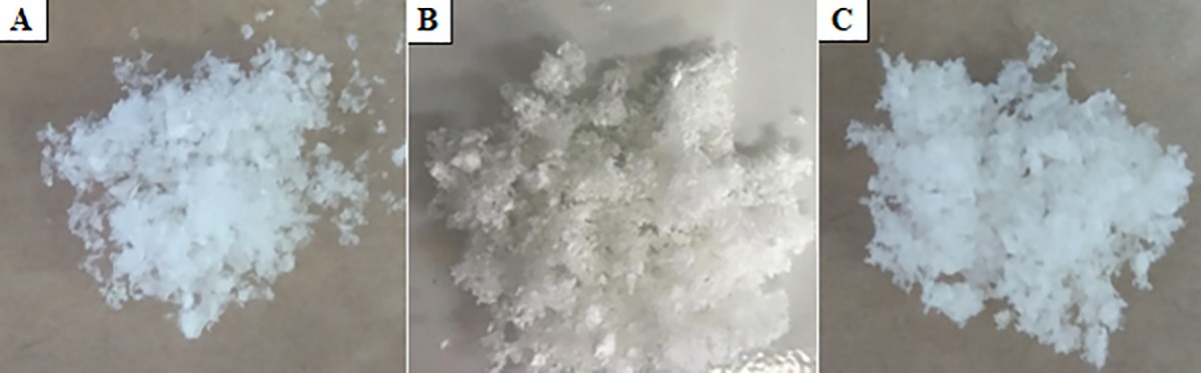
Powder encapsulates containing quinoa oil (*Chenopodium quinoa* Willd.) obtained by O/W emulsification technique. (A) OG: Quinoa oil and porcine gelatin in aqueous phase 2; (B) OWG1: Quinoa oil, whey protein isolate and porcine gelatin in aqueous phase 2; (C) OWG2: Quinoa oil, whey protein isolate in aqueous phase 1, and porcine gelatin in aqueous phase 2.

The most undesirable property regarding the encapsulated powder is the presence of oil on its surface [37]. In the present study, all formulations presented desirable characteristics since there was no oily aspect (Fig 1). Therefore, all particles were submitted for the analyses of physical and chemical characterization.

#### Scanning Electron Microscopy (SEM)

**Fig 2:**
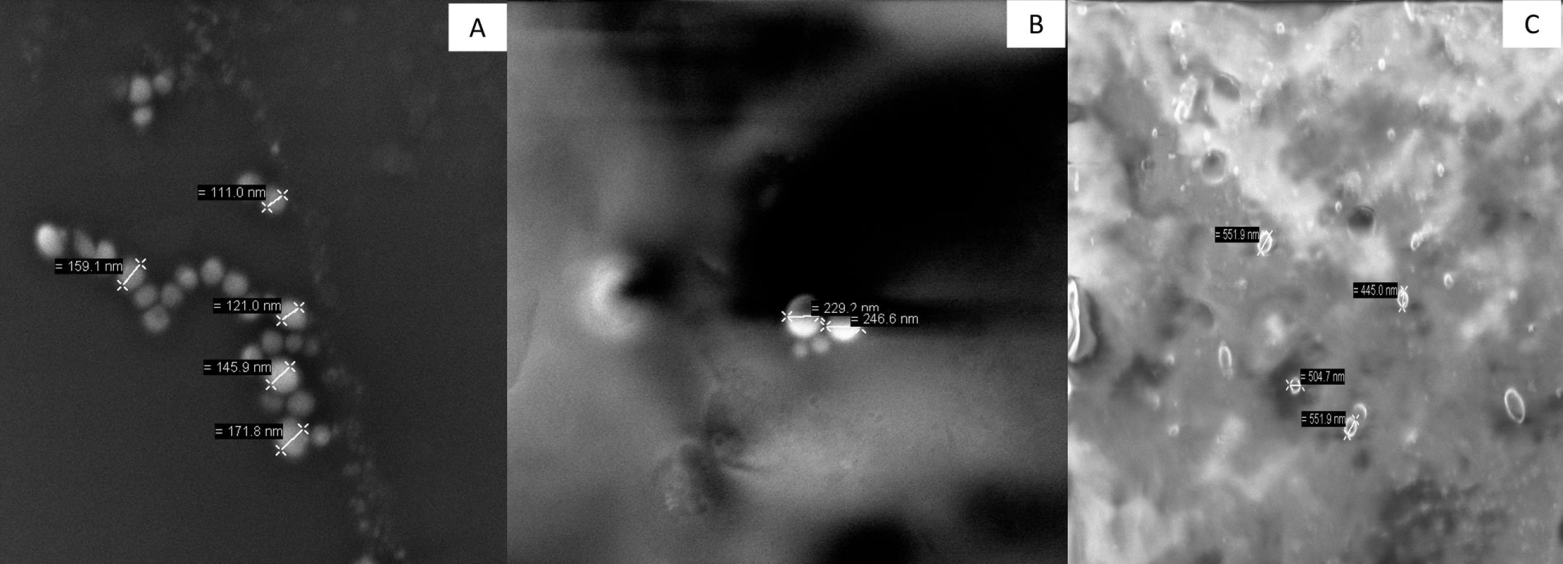
Micrographs of powdered particles, dispersed in acetone, obtained by O/W emulsification technique. (A) OG: Quinoa oil and porcine gelatin in aqueous phase 2; (B) OWG1: Quinoa oil, whey protein isolate and porcine gelatin in aqueous phase 2; (C) OWG2: Quinoa oil, whey protein isolate in aqueous phase 1, and porcine gelatin in aqueous phase 2.

In all the encapsulated, particles presented smooth surfaces, with no depressions, or cracks (Fig 2). Thus, the encapsulated quinoa oil was protected by porcine gelatin and by the combination of whey protein isolate and porcine gelatin for all formulations, which may lead to increased application potential in a food matrix of the encapsulated oil.

Results similar to that found in the present study were observed by other authors when encapsulating different oils and using different techniques. González et al. [20] obtained chia oil encapsulated in isolate soybean protein and maltodextrin by spray-drying and freeze-drying, and verified isolate and also aggregated particles, without pores or cracks.

#### Laser diffraction

**Fig 3:**
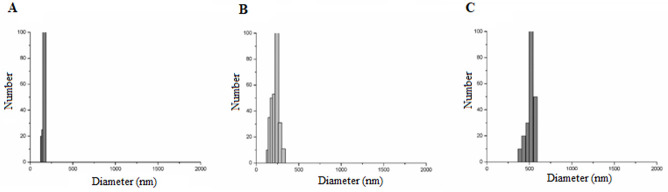
Particle size distribution by laser diffraction of encapsulated powder particles obtained by O/W emulsification technique. (A) OG: Quinoa oil and porcine gelatin in aqueous phase 2; (B) OWG1: Quinoa oil, whey protein isolate and porcine gelatin in aqueous phase 2; (C) OWG2: Quinoa oil, whey protein isolate in aqueous phase 1, and porcine gelatin in aqueous phase 2.

The formulations presented unimodal particle size distribution with a diameter equal to 165.77 (12.74), 238.75 (20.05), and 529.70 (51.90) nm (Fig 3), respectively, for OG, OWG1, and OWG2. Liu at al. (2019) highlight the importance of achieving the nanoscale in one of dimensions (1–100 nm) regarding the application of particles by the food industry.

On the other hand according to the review study by Rezaei et al. (2020) [38], the encapsulates obtained by nanoemulsion have an average size in the range of 50 and 600 nm (Rezaei et al., 2020) [38]. Acosta [39] ports that even nanoparticles in the range of 100–1000 nm are capable of producing a substantial improvement in bioavailability of active ingredients. The researcher demonstrated, through a mathematical model, that the delivery of nanoparticles up to 600 nm would produce an increase in the absorption of vitamin E and that even particles of 1 μm would be sufficiently small to ensure the maximum intake of vitamin K3. Thus, it is essential to analyze, in addition to the particle size, the water dispersibility of the encapsulated compounds produced in the present study to define their application potential aiming the enrichment of the food matrix.

Regarding the polydispersion indices, up to 0.3 indicates that the particle diameter's distribution is in a narrow range. Therefore, they represent the ideal homogeneous diameter of the particles [40]. All formulations tested showed a polydispersion index equal to 0.379 (0.00), 0.618 (0.05), and 0.687 (0.09), respectively, for OG, OWG1, and OWG2. These results indicated their heterogeneity, confirming the SEM results.

A crucial parameter affecting the diameter of the particles is the nature and concentration of the surfactant agent employed in the formulation. The hydrophilicity of a surfactant is measured by its hydrophilic-lipophilic balance (HLB) value, where higher HLB values indicate greater hydrophilicity. The surfactant Tween 20 (HLB = 16.7) [41] was used in the different formulations tested in this study. Based on the literature, this surfactant is an excellent option among polysorbates to produce particles with smaller sizes [42].

Rostami et al. (2018) [43] when encapsulating cumin essential oil, reported an average particle size of 470 nm when using 0.25% of Tween 20 and 126 nm when 1% Tween 20 was used. Thus, an alternative to reduce particle size and obtain a better polydispersion index for the tested formulations would be to increase the concentration of Tween 20 if the size reflected negatively in the oil retention in particles and the water dispersibility.

#### Zeta potential

The Zeta potential result for OG, OWG1, and OWG2 were 12.06 (0.78) mV, 4.52 (0.73) mV, 6.11 (1.28) mV, respectively. The data indicated the presence of positive loads on the surface of all particles.

OG was produced using only the porcine gelatin as an encapsulating agent. Thus, the presence of positive loads was expected, since the pH of the aqueous phases formulated was fixed in 5.5. This pH is below the isoelectric point (pI) of the porcine gelatin (pI = 6 to 9), so OG presented a positive load because the amine groups gained an extra proton [13].

OWG1 and OWG2 were formulated using a combination of porcine gelatin and whey protein isolate as encapsulating agents. The isoelectric point of whey protein isolate varies between 4.8 and 5.3. Therefore, at pH higher than its pI as was the pH of 5.5 used in the emulsions, it presented a negative liquid load because the carboxyl group loses its proton. Based on the positive charges of these particulates, we can infer the predominance of gelatin on the encapsulates surface.

From the Zeta Potential values, it is possible to affirm if the particles are highly unstable (around ± 0–10 mV), moderately stable (± 20–30 mV) and highly stable (above ± 30 mV) [44]. Thus, the particles in the present study are highly unstable in the studied conditions (pH 7.0). Depending on the industrial application desired, a new study of the Zeta Potential is necessary for other pH ranges, in which they may have greater stability.

#### Fourier Transform Infrared Spectroscopy (FTIR)

**Fig 4:**
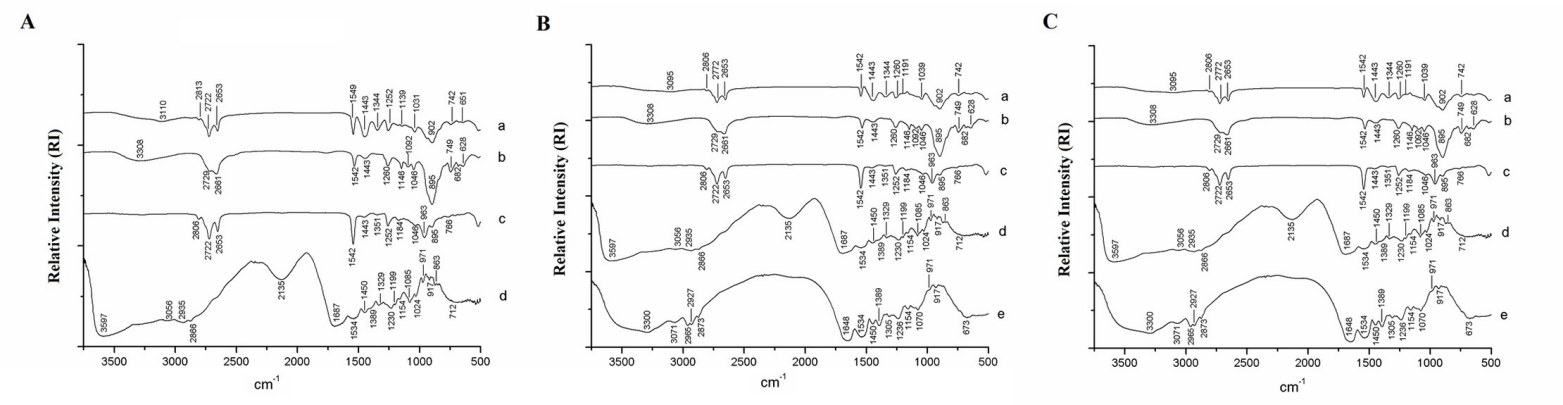
FTIR spectra of powder particles obtained by O/W emulsification technique (A) OG: Quinoa oil and porcine gelatin in aqueous phase 2 –a. OG, b. Tween 20, c. quinoa oil, d. porcine gelatin; (B) OWG1: Quinoa oil, whey protein isolate and porcine gelatin in aqueous phase 2 –a. OWG1, b. Tween 20, c. quinoa oil, d. porcine gelatin, e. whey protein isolated; (C) OWG2: Quinoa oil, whey protein isolate in aqueous phase 1, and porcine gelatin in aqueous phase 2—a. OWG2, b. Tween 20, c. quinoa oil, d. porcine gelatin, e. whey protein isolated.

For the crude quinoa oil spectrum, the vibrational band 2806 cm^-1^ (C–H bond), detected the hydrocarbon groups. The vibration detected in the region 1542 cm^-1^ indicates the strong presence of double bonds (C = C; C = O) in the oil, a characteristic of unsaturated fatty acids (Fig 4).

Considering Tween 20, the presence of the following bands was observed: around 3300 cm^-1^, characterizing the presence of OH bond; at 1542 cm^-1^ referring to C = O bond; and at 895 cm^-1^ associated with the double bond of the methacrylate molecule [45].

In the porcine gelatin spectrum, an absorption range in the region around 3600 cm^-1^ related to the region of O-H bond was observed. A band at 1687 cm^-1^ indicated the presence of the C = O bond (amide I). Another band at 1534 cm^-1^ indicated the presence of a secondary acyclic amide (N-H) [46].

Concerning isolate whey protein, a band at 3300 cm^-1^ was observed, which is characteristic of O-H bond. In addition to this, the following bands were detected: 1648 cm^-1^ referring to the connection C = O, indicative of amide I; 1534 cm^-1^ that corresponds to the C-N elongation of secondary acyclic amide; in the ranges between 1450–1389 cm^-1^ and 1380–1236 cm^-1^ regarding the folding of hydroxyl groups [47].

Regarding OG, it was possible to detect a slight attenuation of the bands 2772 cm^-1^ and 2653 cm^-1^ of crude quinoa oil. The bands 1351 cm^-1^, 1184 cm^-1^, 1046 cm^-1^ and 963 cm^-1^ of the oil were not observed in the encapsulated (Fig 3A). It was also possible to observe the formation of new bands, such as: 1344 cm^-1^, 1031 cm^-1^ and 902 cm^-1^ for OG; and 3354 cm^-1^, 1344 cm^-1^, 1040 cm^-1^ and 947 cm^-1^ for OG. The formation of these new bands is indicative of hydrophobic interactions between the nonpolar amino acids present in gelatin with the carbonic chain of oil. Some displacements of gelatin and oil bands were also observed in the encapsulated, which is also indicative of chemical interactions among the materials present in the formulations. Based on this, the results showed that the quinoa oil was encapsulated by gelatin.

For OWG1 (Fig 3B), attenuations in the 2772 cm^-1^ and 2653 cm^-1^ bands of crude quinoa oil were also observed, slightly more pronounced when compared to the previous formulations. The bands 1252 cm^-1^, 1351 cm^-1^, 1046 cm^-1^, 963 cm^-1^ and 766 cm^-1^ presented in the oil were not observed in OGW1. And the formation of new bands was observed: 3095 cm^-1^, 1344 cm^-1^, 1191 cm^-1^, 1039 cm^-1^ and 902 cm^-1^ for OWG1. Therefore, it is possible to suggest that there are both hydrophobic interactions between the nonpolar amino acids present in gelatin with the hydrophobic region of the whey protein isolate, as it is with the carbonic oil chain.

For OWG2 (Fig 3C), the bands 2772 cm^-1^ and 2653 cm^-1^ of crude quinoa oil presented a more remarkable attenuation than for the other encapsulated ones. The bands 1351 cm^-1^, 1252 cm^-1^, 1184 cm^-1^, 963 cm^-1^, and 766 cm^-1^ present in the oil were no longer found in OWG2. The formation of new bands was observed: 3095 cm^-1^, 1435 cm^-1^, 1336 cm^-1^, 1176 cm^-1^ and 1040 cm^-1^ for OWG2.4. Therefore, for OWG2, it is also possible to suggest that interactions are occurring between gelatin, whey protein isolate, and quinoa oil, protecting even more the quinoa oil.

The results obtained showed that the presence of whey protein isolate was essential in the system to increase the number of chemical interactions between nonpolar amino acids and quinoa oil and, thus, allow for higher entrapment. On the other hand, it was noted that this occurred more efficiently in the formulation in which the encapsulating agents were in separate phases (OWG2). Thus, suggesting that the fraction of oil that did not interact with the nonpolar amino acids of the whey protein isolate, became available to interact with the nonpolar amino acids present in porcine gelatin.

#### X-ray Diffraction (xrd)

**Fig 5:**
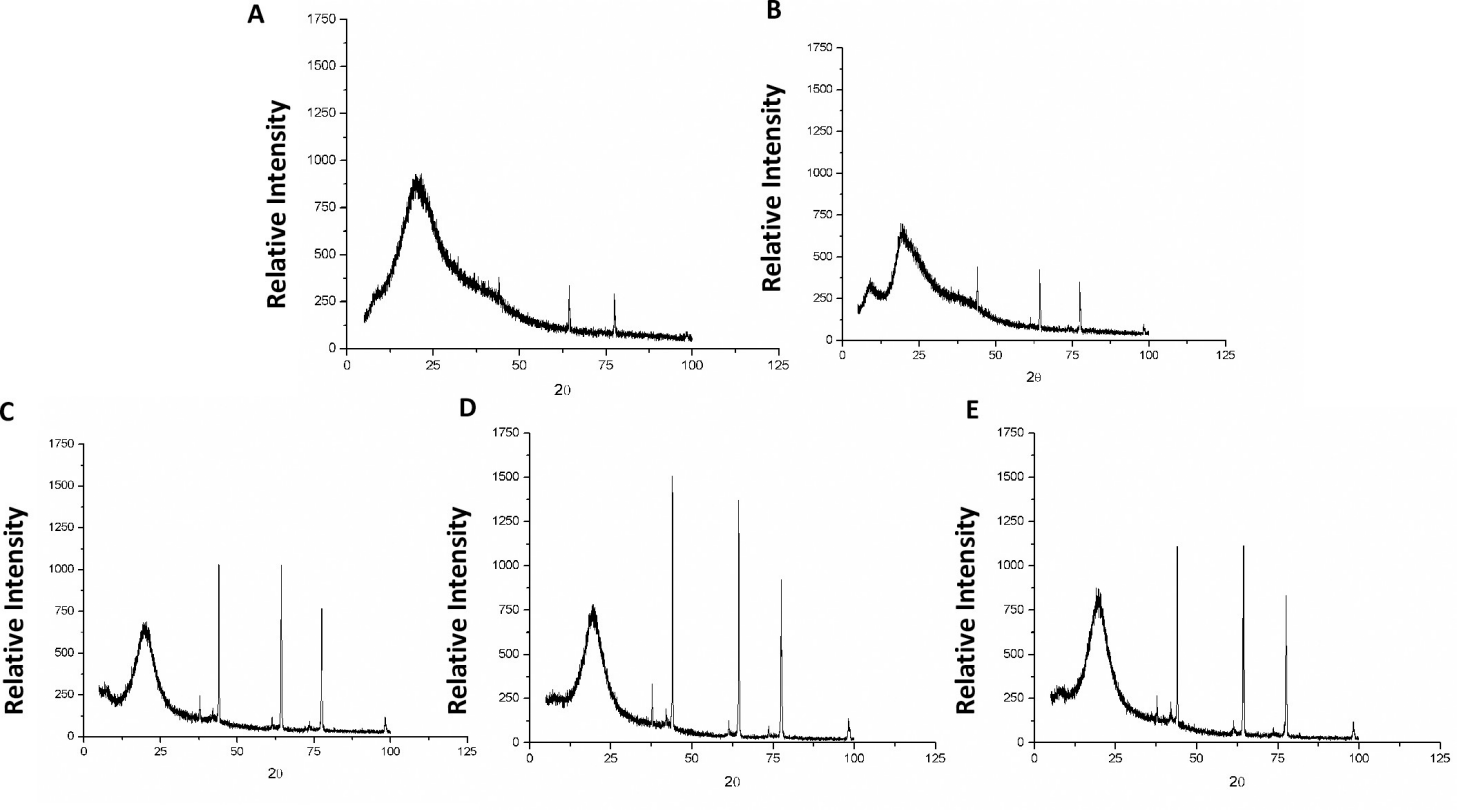
X-ray diffractograms of encapsulating agents and powder particles obtained by O/W emulsification technique. (A) porcine gelatin; (B) whey protein isolated; (C) OG: Quinoa oil and porcine gelatin in aqueous phase 2; (D) OWG1: Quinoa oil, whey protein isolate and porcine gelatin in aqueous phase 2; (E) OWG2: Quinoa oil, whey protein isolate in aqueous phase 1, and porcine gelatin in aqueous phase 2.

The diffractograms of the encapsulating agents and formulates presented semi-crystalline structure, noise background characteristic of amorphous behavior and well-defined peaks that characterize the crystalline regions, as observed in Fig 5.

There was an increase in peaks that characterize the crystalline regions, observed in the encapsulating agent and OG, which can be related to the rise in the amount of quinoa oil, and in the slightly less chemical interaction compared to the other formulations obtained, according to the FTIR results. These results are possibly related to the significant concentration of minerals present in quinoa oil.

OWG1 and OWG2 containing porcine gelatin and whey protein isolate as encapsulating agents, showed attenuation in the intensity of the crystallinity signals when compared with the other formulations. The results indicated that these encapsulates present a better interaction between the encapsulating agents and quinoa oil, confirming the results obtained in FTIR analysis.

#### Quinoa oil (%) present in formulations

Quinoa oil was significantly higher in the formulations OWG1 and OWG2 compared to OG (p < 0,05). The results obtained were 76.9 (1.44), 86.9 (1.89) and 87.5 (3.72), respectively, for OG, OWG1 and OWG2. These results showed that whey protein isolate possibly acted as an extra surfactant load in the system, enabling a higher number of chemical interactions, as observed by FTIR.

According to Taherian et al. [48] the whey protein isolate, due to its hydrophobic and hydrophilic regions, is an essential emulsifier in encapsulations using the emulsification technique, increasing the stability and protection of the active due to a combination of electrostatic and steric interactions. This reflected in the results, which showed that even with an increase in the amount of quinoa oil all systems maintained the same behavior, and the lower amount of quinoa oil was obtained in formulations containing only porcine gelatin (p < 0.05).

#### Water dispersion assay

**Fig 6:**
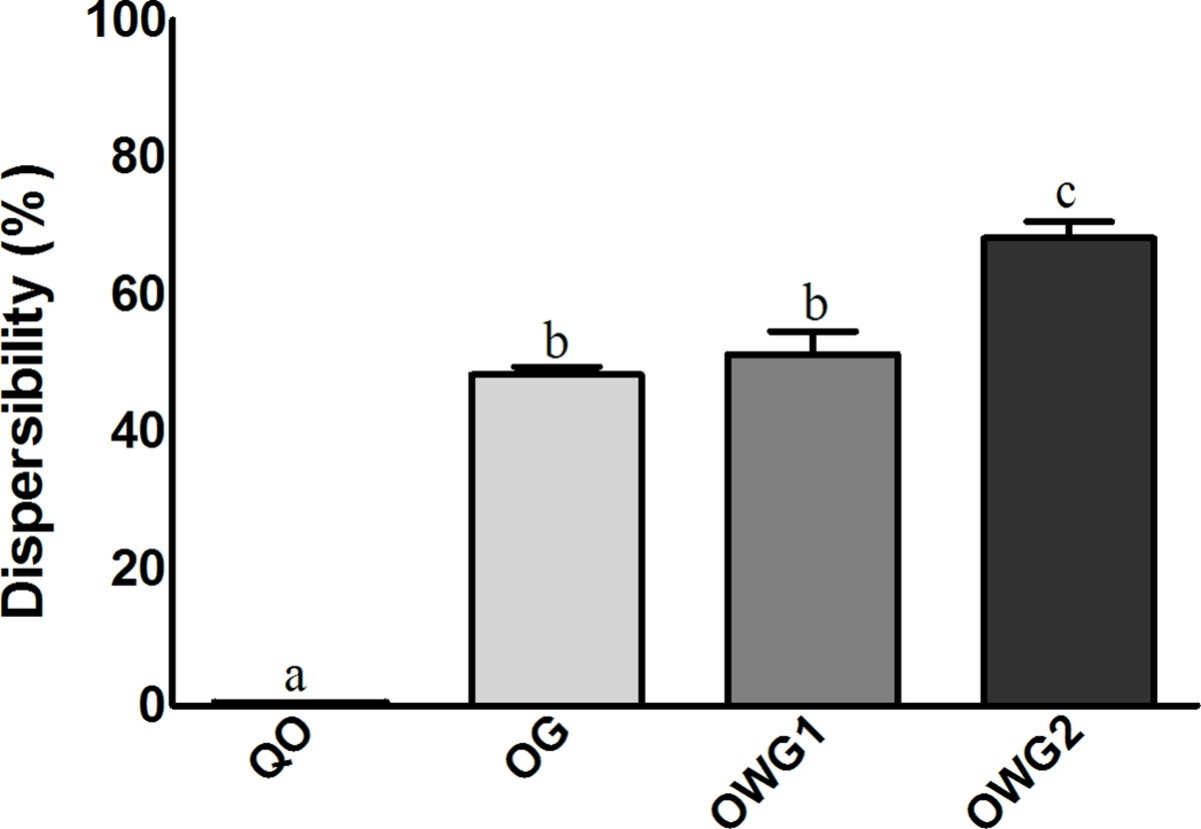
Dispersibility percentages for crude Quinoa Oil (QO) and powder particles obtained by O/W emulsification technique. (A) OG: Quinoa oil and porcine gelatin in aqueous phase 2; (B) OWG1: Quinoa oil, whey protein isolate and porcine gelatin in aqueous phase 2; (C) OWG2: Quinoa oil, whey protein isolate in aqueous phase 1, and porcine gelatin in aqueous phase 2. The values were compared to each other by means of variance analysis with Tukey post-hoc (p < 0.05). *Mean and standard deviation (SD), n = 3. *Equal letters mean that there was no significant difference, according to Tukey post-hoc (p>0.05).

The dispersibility of OG, OWG1, and OWG2 were 48.3% (1.13), 51.3% (43.30), and 68.2% (2.46), respectively (Fig 6).

OG differed significantly from OWG2 (p < 0,05) and presented satisfactory dispersion in water. OWG1 and OWG2 also differ significantly (p < 0,05). Although OWG2 had the largest particle size, a higher percentage of water-dispersion was observed, confirming the previous characterization results that demonstrated the excellent physical and chemical interaction between the encapsulating agents in different aqueous phases (1 and 2) during the processing of the formulations.

As observed in the present study, the encapsulation of quinoa oil led to a significant increase dispersibility. The increase in water dispersion of quinoa oil present in the formulations OG, OWG1, and OWG2 was approximately 14, 15, and 20 times, respectively, compared to non-encapsulated oil.

#### Antioxidant activity

There are numerous techniques to assess bioactive compounds' antioxidant potential [49]. However, previous bench tests showed that the DPPH• radical scavenging method was inefficient for this type of detection using a lipophilic material, compared to ABTS• radical scavenging. This observation corroborates the study by Chuyen et al. (2019).

The IC50 results obtained by the ABTS• assay for QO, OG, OWG1, and OWG2 (Table 3) showed that the quinoa oil presented IC50 values higher than those obtained to evaluated formulations (p < 0.05). This evidences that the encapsulation favored the increase of the antioxidant potential, corroborating with the study of [50].

**Table 3:** Antioxidant activity (IC50) for crude Quinoa Oil (QO) and powder particles obtained by O/W emulsification technique.

Formulation	IC50 (mg/mL)
QO	13.36 (0.28)^a^
OG	8.92 (0.63)^b^
OWG 1	5.30 (0.19)^c^
OWG 2	5.54 (0.27)^c^

OG: Quinoa oil and porcine gelatin in aqueous phase 2; OWG1: Quinoa oil, whey protein isolate and porcine gelatin in aqueous phase 2; OWG2: Quinoa oil, whey protein isolate in aqueous phase 1, and porcine gelatin in aqueous phase 2. The values were compared to each other by means of variance analysis with Tukey post-hoc (p < 0,05).

*Mean and standard deviation (SD), n = 3. The different lowercase (a, b and c) letters indicate a statistical difference (p<0.05). Values with the same lowercase letter are not significantly different, according to Student’s test.

The formulations OWG1 (5.30 mg/mL) and OWG2 (5.54 mg/mL) showed the highest antioxidant activity (p > 0.05), indicating that the form of encapsulation did not interfere with the inhibition of the ABTS radical. Furthermore, it corroborates with the characterization results that showed more significant chemical interaction in formulations based on the combination of whey protein and porcine gelatin. Taktak et al. [51] encapsulated different proportions of fish oil. They observed that the antioxidant activity of the encapsulated was higher than the non-encapsulated fish oil due to the synergistic effect of the proteins used in the encapsulation.

Possibly, these results may be influenced by the higher water-dispersibility observed for the formulations, suggesting that there was an increase in the contact surface for chemical interaction with the water and also with the ABTS radical, resulting in higher radical sequestration and antioxidant activity [52]. This behavior is observed even for OWG2, which presented a larger particle size, but still, according to Acosta [39] it is capable of producing an improvement in bioavailability bioactive compounds.

Therefore, nanotechnology promotes the water dispersibility of lipophilic substances, such as vegetable oils. Besides, it preserves and enhances the biological properties of bioactive compounds in these oils. This can provide many opportunities for the food industry to develop new value-added products or ingredients with market potential [53].

Further studies to evaluate the stability of the formulations in the food matrix to enrich products with unsaturated fatty acids and vitamin E should be conducted. Also, studying the bioactive effect in experimental models aiming to evaluate the protection or promotion of the biological activity of the bioactive constituents in quinoa oil is necessary.

## Conclusion

The studied quinoa oil presented nutritional quality, showing its potential to be used by the food industry. The use of whey protein isolate and porcine gelatin in different aqueous phases of emulsification O/W, and Tween 20 as a surfactant was a promising strategy to make quinoa oil more dispersible in water, and potentialize the antioxidant activity, increasing its potential application in a food matrix.

## Supporting information

S1 Data (XLSX)
